# Structures of multisubunit membrane complexes with the CRYO ARM 200

**DOI:** 10.1093/jmicro/dfac037

**Published:** 2022-07-21

**Authors:** Christoph Gerle, Jun-ichi Kishikawa, Tomoko Yamaguchi, Atsuko Nakanishi, Orkun Çoruh, Fumiaki Makino, Tomoko Miyata, Akihiro Kawamoto, Ken Yokoyama, Keiichi Namba, Genji Kurisu, Takayuki Kato

**Affiliations:** Institute for Protein Research, Osaka University, 3-2 Yamada Oka, Suita, Osaka 565-0871, Japan; RIKEN SPring-8 Center, Life Science Research Infrastructure Group, Sayo-gun, 1-1-1 Kouto, Sayo, Hyogo 679-5148, Japan; Institute for Protein Research, Osaka University, 3-2 Yamada Oka, Suita, Osaka 565-0871, Japan; Graduate School of Frontier Biosciences, Osaka University, Suita, Japan; Department of Molecular Biosciences, Kyoto Sangyo University, Kamigamo-Motoyama, Kyoto 603-8555, Japan; Research Center for Ultra-High Voltage Electron Microscopy, Osaka University, 7-1 Mihogaoka, Ibaraki, Osaka 567-0047, Japan; Institute for Protein Research, Osaka University, 3-2 Yamada Oka, Suita, Osaka 565-0871, Japan; Institute of Science and Technology Austria, Am Campus 1, Klosterneuburg, Niederösterreich 3400, Austria; Graduate School of Frontier Biosciences, Osaka University, Suita, Japan; JEOL Ltd., 3 Chome 1-2 Musashino, Akishima, Tokyo 196-8558, Japan; Graduate School of Frontier Biosciences, Osaka University, Suita, Japan; Institute for Protein Research, Osaka University, 3-2 Yamada Oka, Suita, Osaka 565-0871, Japan; Department of Molecular Biosciences, Kyoto Sangyo University, Kamigamo-Motoyama, Kyoto 603-8555, Japan; Graduate School of Frontier Biosciences, Osaka University, Suita, Japan; RIKEN Center for Biosystems Dynamics Research, 1-3 Yamadaoka, Suita, Osaka 565-0871, Japan; JEOL YOKOGUSHI Research Alliance Laboratories, Osaka University, 1-3 Yamadaoka, Suita, Osaka 565-0871, Japan; Institute for Protein Research, Osaka University, 3-2 Yamada Oka, Suita, Osaka 565-0871, Japan; Institute for Protein Research, Osaka University, 3-2 Yamada Oka, Suita, Osaka 565-0871, Japan; Graduate School of Frontier Biosciences, Osaka University, Suita, Japan

**Keywords:** transmission electron microscope, protein structure, V-ATPase, PSI, molecular motor

## Abstract

Progress in structural membrane biology has been significantly accelerated by the ongoing ‘Resolution Revolution’ in cryo-electron microscopy (cryo-EM). In particular, structure determination by single-particle analysis has evolved into the most powerful method for atomic model building of multisubunit membrane protein complexes. This has created an ever-increasing demand in cryo-EM machine time, which to satisfy is in need of new and affordable cryo-electron microscopes. Here, we review our experience in using the JEOL CRYO ARM 200 prototype for the structure determination by single-particle analysis of three different multisubunit membrane complexes: the *Thermus thermophilus* V-type ATPase V_O_ complex, the *Thermosynechococcus elongatus* photosystem I monomer and the flagellar motor lipopolysaccharide peptidoglycan ring (LP ring) from *Salmonella enterica*.

## Introduction

Membrane proteins and their embedding biomembranes are fundamental to cellular life due to their roles in energy conversion and communication between the inside and outside of the cell [1–4]⁠. In contrast to its importance, our understanding of membrane biology is still rather limited as the dire consequence of the experimental difficulties associated with biomembrane research [5–7]⁠. This circumstance is reflected by the comparatively low number of atomic models for membrane proteins against water-soluble proteins deposited in the Protein Data Bank (PDB): 6238 vs 192 888 (https://pdbj.org/; https://blanco.biomol.uci.edu/mpstruc/), while the share of membrane protein-encoding genes in the human genome is ∼30% [8]⁠.

Since the first direct observation of a transmembrane protein by electron crystallography, bacteriorhodopsin of the purple membrane, in 1975 [9], structures of membrane proteins have been experimentally determined by cryo-electron microscopy (cryo-EM) using image analysis of helical tubes or crystallographic analysis of 2D and 3D crystals imaged by electron and X-ray diffraction [10–14]⁠. However, the challenges in growing well diffracting crystals of to the lipid bilayer adapted membrane proteins puts a severe brake on the structure determination process [15].

The advent of atomic resolution single-particle cryo-EM in the ongoing ‘Resolution Revolution’ [16]⁠ of cryo-EM, a technique that does not require crystals but is able to provide protein Coulomb potential maps at crystallographic resolutions [17–19]⁠, means that this bottleneck of the membrane protein structure determination process is now removed. Moreover, the very recent advances in EM instrumentation of using electron beams with a reduced energy spread by employing either cold field-emission guns (FEGs) [20, 21]⁠ or monochromators [22]⁠ now allow the analysis of well-behaved membrane proteins at a 1.7 Å resolution [21]⁠. These advances in single-particle cryo-EM, however, come at the expense of a steep price tag for state-of-the-art 300 keV cryo-transmission electron microscopy (TEM). Meanwhile, it has been demonstrated for both water-soluble and membrane proteins that when a suitable protein sample is combined with optimized imaging conditions, considerably less expensive 200 keV cryo-TEMs can deliver Coulomb potential maps of sufficient quality for reliable atomic model building [23–26]⁠.

Recently, JEOL has developed a 200 keV cryo-TEM, the CRYO ARM 200, as a relatively affordable instrument for single-particle cryo-EM. In this short review, we describe our experience in analyzing multisubunit membrane protein structures of a eubacterial vacuolar type adenosine triphosphate hydrolase (V-ATPase) [27]⁠, a cyanobacterial photosystem I (PSI) [28] and the lipopolysaccharide peptidoglycan ring (LP ring) of a bacterial flagellar motor [29] using the prototype CRYO ARM 200 (JEOL, Akishima, Tokyo, Japan) operated at a 200 keV acceleration voltage, equipped with a thermal FEG as the electron source, an in-column Ω energy filter, a K2 Summit direct electron detector and the JADAS [30] software and SerialEM [31] for automated data acquisition.

## Membrane protein sample preparation

**Fig. 1.**
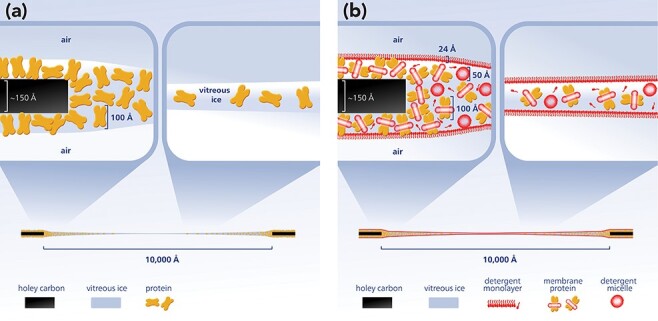
The detergent problem in single-particle cryo-EM. (a) A cartoon of an ideal situation in a single hole of a holey carbon cryo-grid prepared for single-particle cryo-EM imaging. Proteins are randomly oriented in a thin layer of vitreous ice not much thicker than the maximum diameter of the protein. (b) The same situation for DDM solubilized membrane proteins. The presence of detergent in the form of free detergent micelles, free detergent monomers, two free detergent monolayers and membrane protein-bound detergent micelles is depicted in realistic dimensions. Free detergent in the form of two monolayers at the air–water interface complicates high-resolution cryo-imaging by adding a layer of ∼50 Å of carbon and the connected noise to each particle image.

**Fig. 2.**
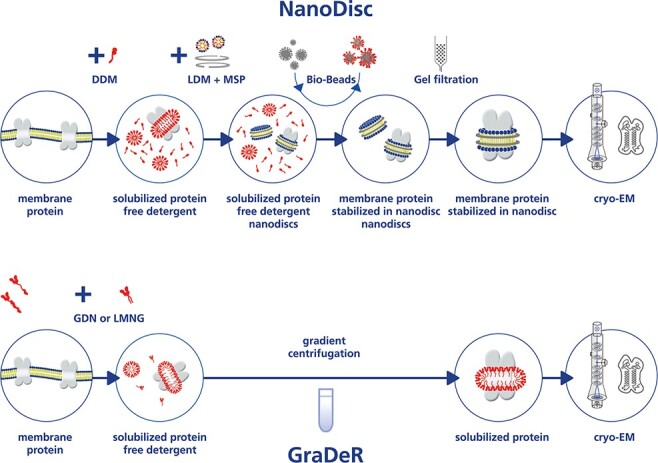
NanoDisc and GraDeR—two solutions to the ‘detergent problem’. (a) Workflow of nanodisc use in single-particle cryo-EM of membrane proteins: after purification of the target membrane protein, the addition of lipids and nanodisc scaffolding protein is followed by BioBeads-mediated detergent removal, leading to the reconstitution of nanodisc-stabilized membrane proteins. After removal of excess nanodiscs by size exclusion chromatography and concentration, the sample is ready for cryo-grid preparation. LDM, lipid detergent micelle; MSP, membrane scaffold protein. (b) GraDeR workflow: solubilization and purification of the target membrane protein in the lipid-like, high-affinity detergents LMNG and GDN or a mixture of them allows the mild and efficient removal of free detergent by density gradient centrifugation. After removal of the crowding agent and concentration, the sample is ready for cryo-grid preparation.

Phase contrast cryo-TEM images of proteins embedded in vitreous ice at electron doses that retain their native structure have a very low signal-to-noise ratio—greatly impacting image processing and 3D reconstruction of high-resolution Coulomb potential maps [32]⁠. The image contrast in cryo-EM images of proteins depends on the difference in density between protein and the embedding vitreous ice (∼1.3 vs ∼1 mg ml^−1^) [33, 34]⁠. Therefore, the use of buffers free of crowding agents, such as glycerol, sucrose and other small organic molecules in single-particle cryo-EM and the formation of a vitreous ice layer only slightly thicker than the target protein imaged, is crucial for the success of a single-particle cryo-EM project [35]⁠. The presence of free detergent in preparations of detergent-solubilized membrane proteins complicates the formation of ideal ice thickness during plunge freezing of cryo-grids. The detrimental effects associated with the presence of detergent can be mainly ascribed to a lowered surface tension, the presence of free detergent micelles in solution and, perhaps most importantly, the formation of detergent monolayers at the air–water interface that might even stack during the blotting and freezing process (see Fig. 1) [36]⁠. This ‘detergent problem’ in single-particle cryo-EM of integral membrane proteins was recognized early on [37]⁠ and in the pioneering work on the transient receptor potential V1 channel (TRPV1 channel) structure, it was successfully solved by replacing the purified channel stabilizing detergent with amphipols [19]⁠. Later studies demonstrated that the reconstitution of membrane proteins into nanodiscs not only eliminates the ‘detergent problem’ but also allows for structure determination of membrane proteins in their physiological environment of the lipid bilayer and in favorable cases the visualization of bound lipids (Fig. 2a) [38]⁠. For membrane proteins recalcitrant to the use of amphipol or nanodiscs, the gradient based detergent removal (GraDeR) approach (see Fig. 2b), combining the novel high-affinity detergents lauryl maltose neopentyl glycol (LMNG) [39] and glycol diosgenin (GDN) [40] with free detergent removal via density gradient ultracentrifugation, has been shown to yield good results [36, 41–43]⁠. This approach is not limited by the diameter of the target protein and as a consequence especially promising for large, fragile multisubunit membrane complexes with a tendency to disassemble in conventional nonionic detergents such as β-dodecyl-maltoside (DDM) [44]⁠. Indeed, in contrast to first-generation nonionic detergents such as DDM, the lipid-like second-generation nonionic detergents LMNG and GDN are much more powerful in retaining structural integrity and functional stability of fragile multisubunit membrane complexes [45, 46]. This is even more true for membrane proteins successfully reconstituted into the lipid bilayer environment of nanodiscs [47]. However, the enhanced stability of the isolated target membrane protein complexes does not guarantee the physiological relevance of any determined structure *per se*. Therefore, the functional analysis of the isolated membrane complexes *in vitro* under conditions that match those that are used for structure determination by single-particle cryo-EM is mandatory. Moreover, since the absence of ligands in the Coulomb potential map can be caused by either physical absence or intrinsic disorder, the actual presence of ligands in the prepared membrane complexes has to be confirmed by other means, e.g. light spectroscopy or mass spectrometry [28]. Finally, in the case of very large membrane complexes containing many polypeptide chains, the use of in situ cryo-electron tomography can be necessary to corroborate the physiological relevance of the overall architecture and oligomeric state of the determined structure. As has been demonstrated nicely for the mitochondrial F-type adenosine triphosphate synthase (F-ATP synthase) [48].

## Structure of the V_o_ domain from *Thermus thermophilus*

Rotary ATPases are evolutionary ancient energy converters essential to the bioenergetics of all cellular life on our planet [49–53]⁠. They are marvelous molecular nano-machines that interconvert electrochemical energy in the form of a transmembrane proton motive force (Δpmf) into the chemical energy of ATP via mechanical rotation [54–58]⁠. Rotary ATPases can be classified into F-type and V-type ATPases. The former is mostly engaged in ATP synthesis, and the latter usually functions as a proton pump. Still, every rotary ATPase is capable of functioning in both directions of the Δpmf—ATP interconversion [59, 60]⁠. V-ATPase from the thermophilic extremophile *Thermus thermophilus* was first isolated from a hot spring in Shizuoka, Japan, and is physiologically working as an ATP synthase [61]. The bipartite division of V-ATPase into the rotary motor V_1_ domain, which harbors the ATPase catalytic sites, and the membrane-spanning V_o_ domain, which harbors the H^+^-transporting rotor ring, is reflected by its assembly pathway ending in a final step of docking V_1_ onto V_o_ [62]_⁠_. Proton tightness of V_o_ in the absence of V_1_ had been demonstrated earlier; however, the structural basis of this change from H^+^ transportation in the V_o_V_1_ holoenzyme versus the auto-inhibited V_o_ form remained unknown.

**Fig. 3.**
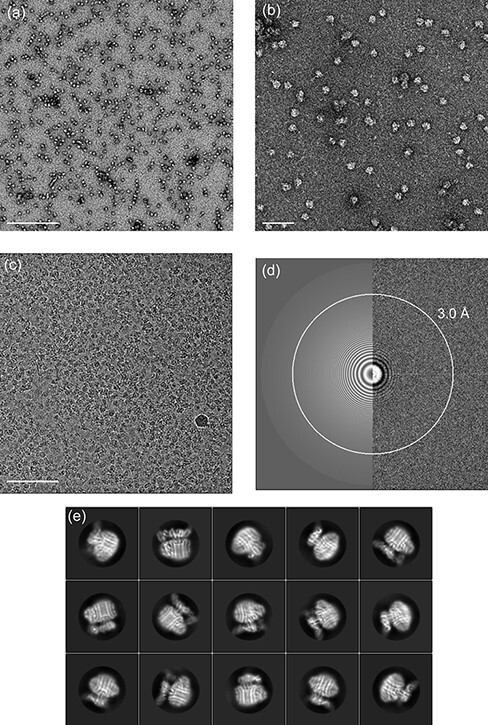
EM image data of the isolated *T. thermophilus* V_o_. (a, b) Electron micrograph of negatively stained isolated V_o_ from *T. thermophilus* reconstituted into nanodiscs exhibiting good monodispersity. Scale bars are 200 nm in (a) and 50 nm in (b). (c) A typical cryo-EM micrograph of the same sample shown in (a) and (b). Scale bar is 100 nm. (d) Fourier transform of the micrograph shown in (c). Thon rings suggest minimal astigmatism. The white circle indicates the maximum CTF resolution of 3.0 Å as estimated by Gctf [108]. (e) Good 2D classes of the isolated V_o_ clearly show secondary structure features of the transmembrane regions. For further information, see Kishikawa *et al.* [27].

**Fig. 4.**
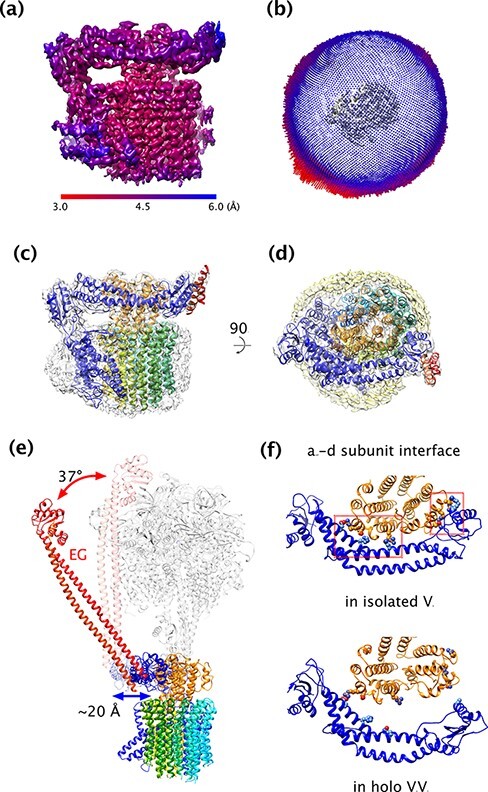
Isolated V_o_: map and model. (a) Local resolution of the final 3D Coulomb potential map of the isolated V_o_ from *T. thermophilus* calculated by ResMap [87]⁠. (b) Euler angle distribution of all images used to calculate the final map; a strong bias toward side views, especially the views as in (a), can be discerned. The isolated V_o_ map in the center is shown from the ‘top’, i.e. cytosolic side. (c) Side view of the final map and final atomic model of the isolated V_o_ in the ribbon style. (d) Top view of (c) from the cytosolic side with the map at a lower threshold and the nanodisc and lipids depicted in yellow. (e) The EG peripheral stalks are swung out from their association with V_1_. The red (upper) and Blue (lower) arrows indicate the conformational changes of the EG peripheral stalk and *a*_sol_, respectively. Subunits in the holo V_o_V_1_ complex are depicted as semi-transparent. (f) The interfaces between the soluble domain of *a* subunit (*a*_sol_) and *d* subunit exhibit large conformational changes between the isolated V_o_ and the V_o_ in holo V_o_V_1_. In the isolated V_o_ complex, the electrostatic interactions indicated by the dashed red boxes inhibit the rotation of the rotor. In contrast, *a*_sol_ swings away from the *d* subunit in the holo V_o_V_1_ complex, releasing these interactions. *a*_sol_ and *d* subunit are colored blue (lower) and orange (upper), respectively. The residues involved in the interactions are represented as spheres. For further information, see Kishikawa *et al.* [27].

Although successful in the description of subcomplex structures [63–68], crystallographic approaches never allowed to visualize the structure of the *T. thermophilus* V_o_V_1_ holoenzyme, nor that of the isolated V_o_ complex. In fact, not a single high-resolution structure of any intact rotary ATPase by crystallographic methods was ever reported. In order to solve the ‘detergent problem’ in single-particle cryo-EM for both V_o_V_1_ and V_o_ complexes, they were first purified in DDM and then reconstituted into 1,2-dimyristoyl-*sn*-glycero-3-phosphocholine lipid nanodiscs via BioBeads-mediated detergent removal and a subsequent final removal of excess scaffold proteins using size exclusion chromatography [27]⁠. The thus prepared V_o_ complexes were applied to freshly 1 min glow discharged holey carbon molybdenum grids (Quantifoil) and plunge frozen (4°C, 100% humidity, Whatman #1, 9 s at blot force 10) in liquid ethane using a Vitrobot Mark IV (Thermo Fisher Scientific). More than 5000 movies were automatically collected with the prototype CRYO ARM 200 (Fig. 3b). The movies were acquired at a nominal magnification of 50 000×, resulting in a pixel size of 1.1 Å, and a defocus range from −1.0 to −3.5 μm with a total of 60 frames for each movie and a total dose of 80 e^−^ Å^−2^. See also Table 1 for a summary of imaging conditions. Images were processed in RELION 3.0 [69, 70] and refined to a final overall resolution [Gold Standard Fourier Shell Correlation (FSC)] of 3.9 Å (Fig. 4a and b). The resulting Coulomb potential map was of sufficient quality for *de novo* atomic modeling of the whole V_o_ complex, and several densities corresponding to lipids could be identified [27]⁠ (Fig. 4c and d). A comparison of the isolated V_o_ structure with that of V_o_ in the context of the V_o_V_1_ holoenzyme indicated almost identical features for the transmembrane region of the rotor ring adjacent to *a* subunit and the rotor ring itself. In contrast, the cytoplasmic domain of *a* subunit *a*_sol_, the *d* subunit and the peripheral stalk subunit E and G (EG) bundle exhibited dramatic conformational changes which together provide a structural basis for the transformation from a proton permissive V_o_V_1_ state of V_o_ to the separated proton tight V_o_ domain (Fig. 4e and f). Unbound from the crown of V_1_, the two EG α-helical bundles are found to swing out from the periphery of the long axis of the V_o_V_1_ complex by 37° (Fig. 4e). The absence of the central stalk subunit from the rotor ring bond *d* subunit together with the released binding of the EG bundles to V_1_ further results in a 45° tilt of helix 6 in subunit *d* and a 15° rotation and a ∼20 Å movement toward the rotor for *a*_sol_. These large-scale conformational changes lead to the formation of electrostatic interactions between *a*_sol_ and the rotor ring bound *d* subunit, effectively blocking futile rotation of the proton transporting C_12_ rotor ring in the isolated V_o_ complex and as a result autoinhibition against proton leaks. Remarkably, structural comparison between prokaryotic and eukaryotic V_o_ suggests the conservation of this molecular mechanism of autoinhibition against proton leaks across species [71–75].

**Table 1.** Imaging conditions for the three multisubunit membrane proteins reviewed here

	Isolated V_o_ PDB 6LY9 EMD-30015	Monomeric PSI PDB ID 6LU1 EMD-0977 EMPIAR-10352	LP ring PDB 7CLR EMD-30398
Molecular weight	290 kDa	330 kDa	10 MDa (basal body)
Microscope	CRYO ARM 200 (JEOL)		
Acc. vol. (kV)	200		
Electron detector	K2 summit (Gatan)		
Total dose (e^− ^Å^−2^)	79.2	80.4	45
Nominal magnification	50 000	60 000	40 000
Calibrated magnification	45 454	56 178	34 482
Pixel size (Å pix^−1^)	1.1	0.89	1.45
Movie frames	60	60	50
Nominal defocus range (μm)	−1.0 to −3.5	−0.5 to −3.5	−0.2 to −2.0
Energy filter slit-width (eV)	10	10	10
Automation software	JADAS (JEOL)		
Resolution (Å)	3.9	3.2	3.5

Imaging conditions chosen for the isolated V_o_ complex are identical to those that were successfully employed for the previous analysis of β-galactosidase at 2.45 Å. For the stable monomeric PSI complex of thermophilic source, we aimed at higher resolution using a relatively higher magnification and an increased lower defocus range. With particle image collection posing a bottleneck, for the analysis of the LP ring in the context of the very large basal body, we chose a relatively low magnification to increase the number of particles in the field of view. Accordingly, the electron dose was lowered. The extreme particle size allowed for further lowering of the defocus range without affecting particle picking.

## Structure of monomeric PSI from *Thermosynechococcus elongatus*

**Fig. 5.**
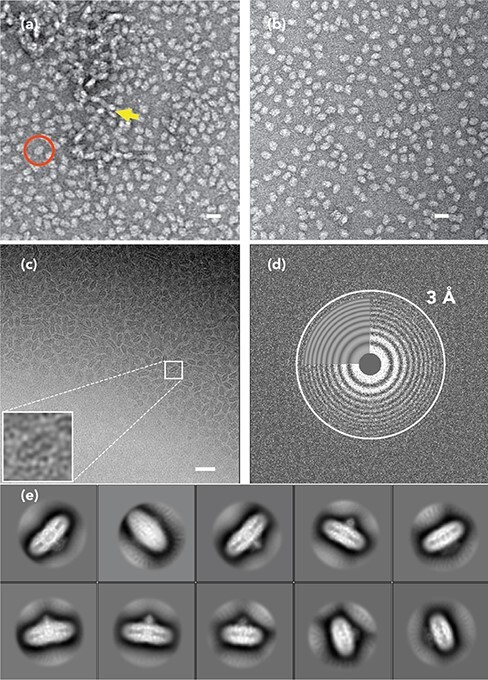
PSI monomer: EM image data. (a) Electron micrograph of purified *T. elongatus* PSI monomers in LMNG after sucrose gradient-mediated removal of free detergent and uranyl acetate negative staining. The PSI monomer exhibited the tendency to form row-like smaller aggregates in both DDM and LMNG. A single PSI monomer is encircled in red, and a row-like aggregate indicated by a yellow arrow. Scale bar, 20 nm. (b) Negative stain EM of the same purification batch of the PSI monomer after performing detergent exchange to GDN and free detergent removal by GraDeR showed good monodispersity. Scale bar, 20 nm. (c) Cryo-EM micrograph of the same sample as in (b). One PSI monomer in side-view orientation along the membrane plane is boxed out and enlarged. Scale bar, 30 nm. (d) Fourier transform of the micrograph shown in (c). Thon rings are visible beyond 3 Å resolution and indicate minimal astigmatism. (e) Good 2D classes of the PSI monomer indicate that side views along the membrane plane of the complex dominate the image data set. In addition, the closeness of neighboring particles is apparent. For further information, see Çoruh *et al.* [28].

**Fig. 6.**
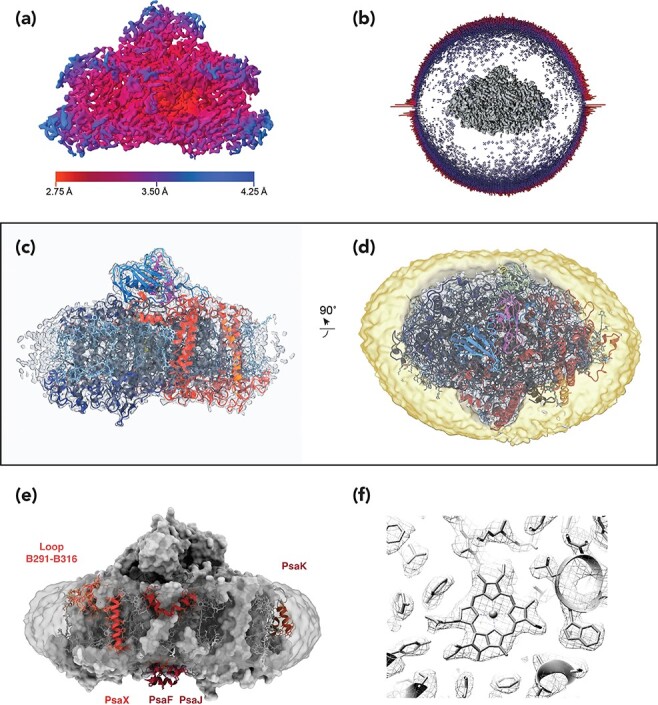
PSI monomer: map and model. (a) Local resolution of the final 3D Coulomb potential map of the *T. elongatus* PSI monomer calculated by ResMap [87] with a resolution better than 3.0 Å in the core. (b) Euler angle distribution of all images used to calculate the final map; length of the columns indicates the relative number of particle images at the local Euler angle position. A strong bias toward side views with an even radial distribution can be discerned. The PSI monomer map in the center is shown from the ‘bottom’, i.e. lumenal side. (c) Side view of the final map and final atomic model of the *T. elongatus* PSI monomer in the ribbon style. (d) Top view of (c) from the stromal side with the map at a lower threshold and the detergent micelle depicted in yellow. (e) Side view from the membrane-facing side with protein as surface, cofactors in gray stick model and the map in transparent gray for the detergent micelle. Protein regions of the *T. elongatus* PSI trimer crystal structure which are disordered in the monomer are depicted in red ribbon and labeled (f) Map in mesh and model in gray stick of a representative chlorophyll exhibiting the typical non-planarity of the chlorine ring. For further information, see Çoruh *et al.* [28].

PSI is nature’s most efficient energy transformer—capable of pumping one electron across the thylakoid membrane for each photon absorbed by its pigment network of carotenoids and chlorophylls [76]. The 2.5 Å crystal structure of the *T. elongatus* PSI trimer reported in 2001 was the first atomic description of this multisubunit membrane protein central to the light reactions of oxygenic photosynthesis [77]. However, although isolation, spectroscopic characterization and crystallization of its monomeric form were already described in the 1990s [78]⁠, the high-resolution structure of the *T. elongatus* PSI monomer remained unknown. A striking difference between cyanobacterial PSI and that of algae and higher plants is that cyanobacterial PSI is present in oligomeric forms, mostly trimers, whereas algal and plant PSI is strictly monomeric and is always connected to an outer antenna of light-harvesting complexes [79–81]⁠. Possibly related to this difference in oligomeric organization is the observed dependence of ‘red’ chlorophyll absorption on the oligomeric state in cyanobacterial PSI [82]⁠. In contrast, plants and algae outsource ‘red’ chlorophyll activity to the light-harvesting complexes of the outer antenna of PSI [83]⁠. The biological role of ‘red’ chlorophylls is to allow the productive use of light with wavelengths beyond that of the reaction centers absorbing wavelength by using thermal energy as a means to bump up the excitation energy for transfer to an open reaction center. Additionally, in the case of a closed reaction center, ‘red’ chlorophylls allow the quenching of dangerous excess excitation energy by its transfer to a thermal energy sink. Although the loss of ‘red’ chlorophyll absorption in *T. elongatus* PSI upon monomerization is spectroscopically well documented, the absence of an atomic model for the monomer left their location among the total of 96 chlorophylls obscure [84]. A newly developed purification method for *T. elongatus* PSI in its monomeric form allowed us to establish a preparation of intact and fully functional PSI monomer in the milligram range, i.e. suitable for structural studies by single-particle cryo-EM. The examination of the purified complex by negative stain EM showed the tendency of *T. elongatus* PSI monomers to form small row-like aggregates in the presence of the detergents DDM and LMNG [39] (Fig. 5a). Switching to the novel, high-affinity detergent GDN [40] greatly enhanced monodispersity (Fig. 5b)—removing this obstacle for structure determination by single-particle cryo-EM. The ‘detergent problem’ was solved using the GraDeR approach for the removal of excess free detergent in an additional sucrose density gradient step. GraDeR prepared PSI monomers were concentrated to 7.5 mg ml^−1^, and sucrose was removed using centrifugal concentrators (AMICON, molecular weight cut-off: 100 kDa). The almost complete absence of free detergent micelles, easily discernible in the background of uranyl acetate stained specimens, and good monodispersity were evaluated by negative stain EM (Fig. 5b). For cryo-EM imaging, in a first and at the same time also final trial, a volume of 2.6 μl of PSI monomers was applied to a total of three Quantifoil holey carbon copper grids that had been freshly glow discharged on both sides for 60 s, blotted and flash frozen in liquid ethane using a Vitrobot Mark IV (Thermo Fisher Scientific) (4°C, 95% humidity, Whatman #1, 3 s at blot force 0) and transferred to the prototype CRYO ARM 200. To judge ice thickness, we took advantage of the energy filter by comparing average pixel values in filter ‘on’ and ‘off’ modus. Areas exhibiting ratios of 0.85 were judged as being suitable for a ∼3.0 Å reconstruction and marked for automatic data acquisition. Regions of good ice thickness had particles densely packed to the edge of the hole with a clear tendency for preferred side views (Fig. 5c)—aspects of cryo-grid quality in this study that clearly deserve improvement in future studies. A total of 1530 movies were acquired using the prototype CRYO ARM 200 at 60 000× nominal magnification corresponding to a pixel size of 0.89 Å. Each movie was taken at 12 s exposure containing 60 frames with an electron dose of 1.34 e^−^ Å^−2^ per frame resulting in a total dose of 80.4 e^−^ Å^−2^ and a defocus range from −0.5 to −3.5 μm with one stage position for each acquired movie (Table I). With the exception of *ab initio* structure calculation, which was performed in cryoSPARC [85, 86]⁠, all image processing was performed in RELION 3.0 [69, 70]. Even though the densely packed particles showed a strong propensity for side views along the membrane plane, the good radial distribution of Euler angles (Fig. 6b) in the final data set of 46 105 particle images allowed successful 3D auto-refinement. Extensive rounds of contrast transfer function (CTF) refinement and motion correction at the single-particle level using Bayesian polishing gave a Coulomb potential map with a final overall resolution (Gold Standard FSC) of 3.2 Å and a local resolution of 2.75 Å (estimated using ResMap [87]) at the PSI core (Fig. 6a). Model building in Coot [88] and Phenix [89] was performed employing the in 2001 published X-ray crystal structure of the *T. elongatus* trimer (PDB ID: 1JB0 [77]⁠) as a starting model, resulting in a final atomic model of the PSI monomer with very good refinement statistics. Almost half of all atoms in PSI are those of cofactors, therefore the use of accurate cofactor restraint files was instrumental for successful refinement of the atomic model (Fig. 6f). For chlorophyll *a* and β-carotene, these were calculated using the Grade Server (http://grade.globalphasing.org) [90],⁠ whereas restraints for the cubic [Fe_4_S_4_] iron–sulfur clusters were from Moriarty and Adams [91]⁠. The comparison of our atomic model of the *T. elongatus* PSI monomer (Fig. 6c and d) with that of the *T. elongatus* PSI trimer allowed us to find important structural differences that can explain the loss of long wavelength chlorophyll absorption by the disordering but not loss of a chlorophyll cluster in the membrane-facing region of the PsaX subunit (Fig. 6e). Quantifying the relative disorder or mobility of cofactors is useful for structural evaluation of individual or groups of cofactors in photosystems that can harbor hundreds of pigments involved in the absorption and transfer of excitation energy. The recently published *Q*-scores [92]⁠ available as a Chimera [93]⁠ plug-in provide a convenient means to quantify the resolvability of each single atom in a model fitted into a given Coulomb potential map. Importantly, for photosystems, this creates the possibility to compare relative disorder of individual pigments and might be especially useful in higher resolution maps of photosystems to clarify the relationship of pigment mobility and spectroscopic behavior. Our structure-based location of cyanobacterial ‘red’ chlorophylls at a membrane-facing cavity on the periphery of the PsaB subunit allowed us to propose three new hypotheses [28]. First, lipids act as a vibrational energy source or sink depending on the reaction center’s needs. Second, cyanobacterial PSI oligomerization serves as a molecular switch for the activity of ‘red’ chlorophylls. This notion implies that during the evolution of PSI, oligomerization became redundant for algal and plant PSI as a consequence of their ‘red’ chlorophylls being outsourced to the light-harvesting complexes. Third, the peripheral location of excitation energy trapping ‘red’ chlorophylls at the far side of cyanobacterial PsaB is underlying the less frequent use of the cyanobacterial PsaB electron transfer chain branch compared to the nearly equal use of both branches in PSI of higher plants.

## Structure of the LP ring from *Salmonella enterica*

Bacteria such as *Salmonella* swim in solution using a rotary motor called the flagellar motor. The bacterial flagellum is divided into three main elements: the filament acting as a propeller, the hook acting as a universal joint and the basal body generating torque for filament rotation [94, 95]. The flagellar basal body shares structural similarity to artificial rotary motors and consists of four active components: C ring, MS ring, LP ring and the rod. The MS-C ring acts as a rotor and is surrounded by the stator complexes that channel the flow of protons from the outside of the inner membrane to the cytoplasm and convert it into torque—driving the rotor with ∼100% efficiency. The LP ring consists of a lipoprotein, FlgH and a periplasmic protein, FlgI, that together form a cylindrical structure that surrounds the rod as a drive shaft. The LP ring is embedded in both the outer membrane and the peptidoglycan layer and acts as a bushing [94, 95]. The length of the flagellar filament is up to 10 times longer than that of the cell, and the flagellar motor can rotate it at speeds of up to 1700 rpm against the viscous resistance of water [96]. As a result, the LP ring requires a mechanism to support the rod for its stable rotation while keeping friction minimal. For understanding of how the LP ring acts as an efficient bushing, we used cryo-EM to determine its high-resolution structure.

**Fig. 7.**
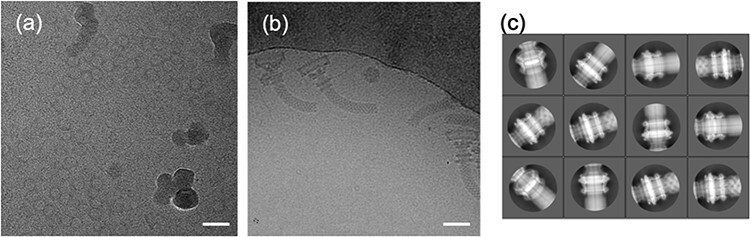
EM image data of the LP ring and hook-basal body isolated from *S. enterica*. (a) A representative cryo-EM micrograph of the isolated LP ring. (b) A representative cryo-EM micrograph of the hook-basal body. (c) Good 2D classes of the LP ring portion of the hook-basal body, including part of the hook and rod. Since the LP ring was focused in 2D classification, the structure of the LP ring is clearly seen, whereas that of the rod and hook is obscure. Scale bars, 40 nm. For further information, see Yamaguchi *et al.* [29].

Since the purification of the LP ring has been already established [97, 98], we initially attempted to perform structural analysis on the purified LP ring alone. For this purpose, we purified the LP ring from *Salmonella enterica* serovar Typhimurium, however, initially only at very low yield. In addition, due to the tendency of LP ring particles to adsorb onto the carbon film, collecting a sufficient number of particle images proved to be challenging. To solve these problems, we first used genetic engineering to increase the number of flagella motors per cell and also performed cell culturing at much larger volumes. This enabled us to freeze cryo-grids exhibiting a sufficient number of particles in the field of view; still, 3D reconstruction was not possible due to the severely preferred end-on orientation of the LP ring complexes (Fig. 7a). To overcome these challenges, we decided to, instead of the isolated LP ring, use the complete hook-basal body for our analysis of the LP ring structure.

The hook-basal body was isolated using Triton X-100, an inexpensive detergent that is used for hook-basal body purification since the early days of bacterial flagellar research [97]. Triton X-100 turned out to be sufficient both for purification and also high-resolution cryo-EM imaging of the hook-basal body, which is likely a consequence of its large supramolecular structure with a molecular mass of >10 MDa, effectively diminishing the detrimental influence of free detergent.

For structure determination by single-particle cryo-EM, the purified hook-basal body was applied to freshly glow discharged Quantifoil holey carbon grids and plunge frozen in liquid ethane using a Vitrobot Mark IV (Thermo Fisher Scientific). Cryo-EM images were recorded using the prototype CRYO ARM 200. A total number of 12 759 movies, each containing 50 frames, were collected with a total electron dose exposure of 45 e^− ^Å^−2^. All movies were recorded at a nominal magnification of 40 000× corresponding to a pixel size of 1.45 Å, using a defocus range from −0.2 to −2.0 μm (Table I).

Particle picking proved to be challenging. First, the number of particles in the field of view was relatively small; second, particles tended to crowd near the carbon edge (Fig. 7b) and third, the non-spherical, asymmetric shape of the particle made it difficult to detect its center and prevented the use of standard automatic picking procedures. We improved the efficiency and accuracy of particle identification by developing a particle picker program, YOLOPick, based on a convolutional neural network program, YOLO [99]. After picking and extracting a sufficient number of particle images, 2D classification was carried out, and only good 2D class averages were selected for further processing (see Fig. 7c). The image signal corresponding to the hook-basal body other than the LP ring was subtracted from all images for high-resolution refinement of the LP ring structure. All image analysis was performed using RELION 3.0-beta [69, 70], and the final LP ring structure was successfully solved at an overall resolution (Gold Standard FSC) of 3.5 Å from a total of 10 802 particles. Importantly, the resulting Coulomb potential map was of sufficient quality for *de novo* atomic modeling.

**Fig. 8.**
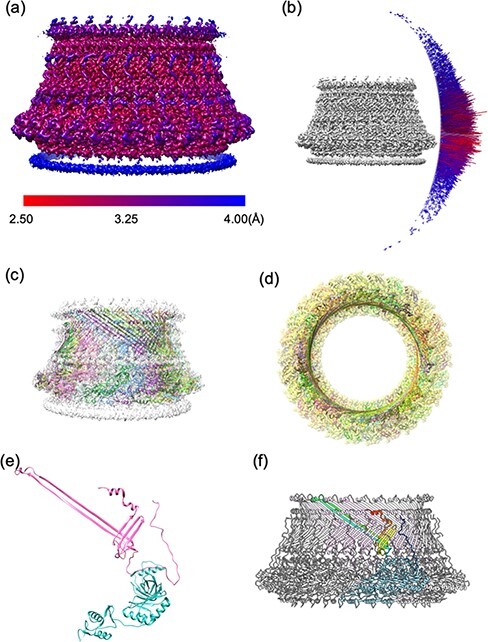
LP ring: map and model. (a) Local resolution of the final 3D Coulomb potential map of the LP ring calculated by blockres in the Bsoft package [109]. (b) The Euler angle distribution of all images is used to calculate the final map. Since the hook-basal body is not easily oriented end-on in ice, it shows a strong bias toward side views. (c) Side view of the final map with the final atomic model depicted in the ribbon style. (d) Top view of map and model from the outside of the cell. (e) The atomic models of FlgH and FlgI colored in pink (upper model) and cyan (lower model), respectively. FlgH contains two long β-strands and three β-strands that are orthogonal to each other. (f) The atomic model of the LP ring. One FlgH subunit (rainbow colored) interacts with six neighboring FlgH subunits (pink) and three neighboring FlgI subunits (cyan), thereby maintaining the stable ring structure. For further information, see Yamaguchi *et al.* [29].

The LP ring forms a cylinder with an inner diameter of 135 Å, an outer diameter of 260 Å and a height of 145 Å and exhibits a 26-fold rotational symmetry (Fig. 8). Atomic models of FlgH and FlgI were built using Coot [88] and Phenix [89] (**Fig. 8c****–****f**). FlgH forms the L ring wall with a three-layer structure. The inner layer contains two very long antiparallel β-strands and a short β-strand with an α helix. The middle layer contains four antiparallel β-strands with two short α helices. The third, outer layer consists of an extended chain covering the inner two layers. The long antiparallel β-strands of the inner layer and the three stranded β-sheet of the middle layer domain are crossing nearly perpendicular to each other. The long β-strands of the inner layer domain and the three stranded β-sheet of the middle layer domain interact with up to six neighboring FlgH molecules in the LP ring, indicating that such complex and intimate interactions between FlgH subunits are responsible for the mechanical and chemical stability of the LP ring [29] (Fig. 8e and f).

## Conclusion and prospects

In summary, we have demonstrated that even the prototype of CRYO ARM 200 equipped with a conventional thermal FEG, an in-column Ω energy filter, a K2 Summit direct electron detector and the JADAS software for automatic data collection is able to serve as more than a mere screening machine. And that it is capable, when combined with suitable sample preparation and image analysis, to produce high-resolution single-particle cryo-EM structures of multisubunit membrane proteins that can advance our understanding of membrane biology. In all three projects described here, we used JADAS for automatic data collection with one movie taken for each stage position. This limits the number of movies taken per day to around 1000. In contrast, the commercial version of the CRYO ARM 200 using SerialEM for beam-shift-based data acquisition allows routinely acquisition of up to 25 movies per stage position, effectively resulting in a >10-fold increase in data acquisition speed [100]. This increase in speed, which can be even accelerated further by taking multi-shots per hole [101, 102], means that sufficient amounts of data can be taken within a single day for most single-particle analysis projects. With Thon rings in images of thin films of amorphous platinum–iridium extending up to 1.8 Å, the spatial resolution limit of the thermal FEG-equipped prototype CRYO ARM 200 is comparable to that of the XFEG-equipped Titan Krios (Thermo Fisher Scientific) [103]. The implementation of cold FEG technology in the commercial CRYO ARM 200 reduces the energy spread of the electron beam from 0.8 to 0.4 eV—significantly enhancing the signal in the better than 2.0 Å resolution range [26]. In addition, several improvements were made in the newly released ‘CRYO ARM 200 II’ (JEM-Z200CA). These include fringe-less imaging by Köhler illumination and a narrower gap pole piece, which allows for a spherical aberration coefficient (*C*_s_) of 1.5 mm and a chromatic aberration coefficient (*C*_c_) of 1.8 mm that together should boost the achievable resolution. However, further improvements in both software and hardware of the commercial CRYO ARM 200 are desirable. SerialEM is a powerful software, but it is also relatively complex to use. For single-particle analysis projects, user friendliness in data acquisition could be improved through a specialized graphical user interface (GUI) for SerialEM or newer versions of JADAS that include beam-shift-based data acquisition strategies. Furthermore, higher stability of the in-column Ω energy filter enabling the routine use of slit-widths narrower than 10 eV would be beneficial. Also, setting the slider adopter cartridge, which is used for the transfer of auto-grids (Thermo Fisher Scientific), as the standard type of cartridge also for bare cryo-grids could simplify the ‘clipping’ of cartridges considerably. Finally, future versions of the cartridge-based cryo-grid transfer system of the CRYO ARM 200 should make it practical to recover cryo-grids after their examination. Together with the improvements suggested above, as well as others not yet conceived or voiced, the CRYO ARM 200 has the potential to develop into a standard analytical instrument for the biomedical sciences. This is likely contingent, however, on the reduction of the price in order to meet the budgetary requirements of a broad range of future users. This might spark a democratization of cryo-EM image data collection that matches the democratization initiated for image processing by the development of graphics processing units (GPU)-accelerated computing [70, 104]. Together with recent breakthroughs in membrane protein structure prediction [105–107], this hopefully enables the emergence of structural membrane biology at a level that rivals structural biology of soluble proteins.
