# Correction to: Structures of multisubunit membrane complexes with the CRYO ARM 200

**DOI:** 10.1093/jmicro/dfae057

**Published:** 2024-12-26

This is a correction to: Christoph Gerle, Jun-ichi Kishikawa, Tomoko Yamaguchi, Atsuko Nakanishi, Orkun Çoruh, Fumiaki Makino, Tomoko Miyata, Akihiro Kawamoto, Ken Yokoyama, Keiichi Namba, Genji Kurisu, Takayuki Kato, Structures of multisubunit membrane complexes with the CRYO ARM 200, *Microscopy*, Volume 71, Issue 5, October 2022, Pages 249–261, https://doi.org/10.1093/jmicro/dfac037

An important acknowledgment was omitted in the original publication of the article. The acknowledgment section should read as follows:

We would like to express our gratitude to Meltem Tatli for providing the cryo-EM image of the isolated LP ring. And we are grateful for initiation and scientific support from Matthias Rögner, Marc M. Nowaczyk, Anna Frank and Yuko Misumi for the PSI monomer project and also would like to thank Hideki Shigematsu for critical reading of the manuscript. And we are indebted to the two anonymous reviewers who helped us to improve our manuscript.

The authors express sincere apologies to Dr. Meltem Tatli.

